# Electric field detection as floral cue in hoverfly pollination

**DOI:** 10.1038/s41598-021-98371-4

**Published:** 2021-09-21

**Authors:** Shahmshad Ahmed Khan, Khalid Ali Khan, Stepan Kubik, Saboor Ahmad, Hamed A. Ghramh, Afzal Ahmad, Milan Skalicky, Zeenat Naveed, Sadia Malik, Ahlam Khalofah, Dalal M. Aljedani

**Affiliations:** 1 Laboratory of Apiculture, Department of Entomology, Pir Mehr Ali Shah Arid Agriculture University, Rawalpindi, 46000 Pakistan; 2 Research Center for Advanced Materials Science (RCAMS), King Khalid University, P.O. Box 9004, Abha, 61413 Saudi Arabia; 3 Unit of Bee Research and Honey Production, Faculty of Science, King Khalid University, P.O. Box 9004, Abha, 61413 Saudi Arabia; 4 Biology Department, Faculty of Science, King Khalid University, P.O. Box 9004, Abha, 61413 Saudi Arabia; 5 Department of Zoology and Fisheries, Faculty of Agrobiology, Food, and Natural Resources, Czech University of Life Sciences Prague, Kamycka 129, 165 00 Praha 6-Suchdol, Czech Republic; 6 Institute of Apicultural Research/Key Laboratory of Pollinating Insect Biology, Ministry of Agriculture, Chinese Academy of Agricultural Sciences, Beijing, 100093 China; 7 Department of Physics, Allama Iqbal Open University, Islamabad, 44000 Pakistan; 8 Department of Botany and Plant Physiology, Faculty of Agrobiology, Food, and Natural Resources, Czech University of Life Sciences Prague, Kamycka 129, 165 00 Prague, Czechia; 9 Department of Botany, University of Gujarat, Gujarat, 50700 Pakistan; 10 Department of Biotechnology, Fatima Jinnah Women University, Rawalpindi, Pakistan; 11 Department of Biological Sciences, College of Science, University of Jeddah, Jeddah, Saudi Arabia

**Keywords:** Ecology, Physiology, Zoology

## Abstract

Pollinators can detect the color, shape, scent, and even temperature of the flowers they want to visit. Here, we present the previously unappreciated capacity of hoverflies (*Eristalis tenax* and *Cheilosia albipila*) to detect the electric field surrounding flowers. Using hoverflies as key dipteran pollinators, we explored the electrical interactions between flies and flowers—how a hoverfly acquired a charge and how their electrical sensing ability for target flowers contributed to nectar identification and pollination. This study revealed that rapid variations in a floral electric field were related to a nectar reward and increased the likelihood of the fly’s return visits. We found that thoracic hairs played a role in the polarity of hoverfly charge, revealing their electro-mechanosensory capability, as in bumblebees (*Bombus terrestris*). Electrophysiological analysis of the hoverfly’s antennae did not reveal neural sensitivity to the electric field, which favors the mechanosensory hairs as putative electroreceptive organs in both species of hoverflies.

## Introduction

Floral cues such as shape, color, and odor are produced by flowers to attract pollinating insects for their reproduction^1,2^. These cues are the main discriminators that allow pollinators to select suitable flowers for pollen and nectar collection^3,4^. The floral cues most commonly influencing pollinators’ behavior are color, pattern, fragrance, petal arrangement, presence or absence of ‘fingerprints’ of previous insect visitors, temperature, and humidity in the area^2,5^. Since Aristotle, a variety of studies have been carried out to determine the effect of these cues on pollinators’ behavior, but so far the exact mechanisms for detection and decision making remain elusive^2^. Diversity in floral cues increases a plant’s attractiveness to pollinators and consequently the chances of pollination and ultimately reproductive success^6^.

Electrostatic charges carried by both biotic and abiotic factors play a role in pollination^7^. The probability of the adherence of electrically charged pollen grains to susceptible stigmas is higher than that of uncharged grains. The atmospheric electrical potential gradient has been proposed to play a key role in the electrostatic interactions between pollen and floral reproductive organs^8^. Previous studies showed that an insect’s body can also accumulate an electric charge^9,10^. Like many other insects, pollinators such as honeybees and bumblebees usually develop a positive charge^11–13^. In contrast, most flowers exhibit a negative charge^14–17^. The difference in electrostatic polarity between positive insects and negative flowers generates an attractive force that facilitates the transfer of pollen grains^15^. The positive charge on the surface of an insect’s body can result from friction during flight or walking on a surface^10,15^. Consequently, positively charged insects may significantly affect the transfer of pollens during floral visits^16^. For example, pollinators touched with a charged metallic rod attract more pollen than uncharged insects^18^.

Bumblebees, honeybees, and hoverflies are the most important pollinators of agricultural crops; but, along with other families of flies, the role of hoverflies in plant pollination is underappreciated^19^. The Diptera often make up a similar proportion of the flower-visiting insects as the Hymenoptera, and even dominate in some cases as at higher altitudes^20^. Hoverflies are considered one of the most important pollinators of wild plant species^21,22^, even in some cases as necessary as bees^23^. Hoverflies are also crucial for the pollination of a large number of crops^24–28^, but they have received relatively little attention from scientists.

Electroreception is the ability of an insect to detect weak electric fields (e-fields) in the external environment. It is well known that some fishes and amphibians, and the platypus can detect weak e-fields in their environments^29^. But in recent years, electroreception under aerial conditions mainly by pollinating insects, such as bees has been hypothesized, investigated and demonstrated^15,30^. Honey bee, *Apis mellifera*^31^ and bumblebee, *Bombus terrestris*^13^ have shown the ability to detect weak e-fields while flying, by using specific sensory organs^30^. In the complex world of plant-pollinator interaction any cue that increases pollination efficiency of pollinators should be mutually beneficial. So far, there have been no studies on hoverflies, their putative electrostatic charge, and the possible role of electrostatics in their pollination ability. Here, we investigated the putative sensitivity of the hoverflies, *Eristalis tenax* and *Cheilosia albipila*, to weak electrostatic fields, and documented the role of electrostatics in the behavioral interaction between hoverflies and flowers, including flower surveillance.

## Results

**Figure 1:**
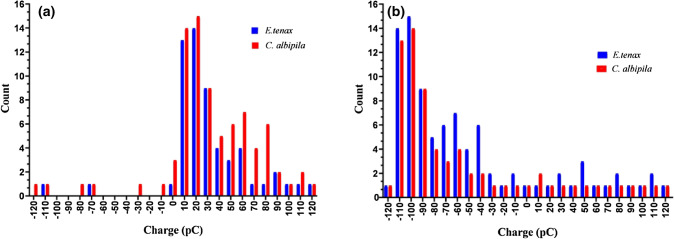
The electric charge carried by both species and it's transfer to artificially prepared flowers. The histogram of charge carried by flying hoverfly species; measured by the Faraday pail instrument. (**a**) *Cheilosia albipila* carried a relatively higher charge than the *Eristalis tenax* and charge (pC) changes into positive when the flies enter into the Faraday pail and shouts up when it touches the flowers. (**b**) Indicates the net opposite charge on both species after removing thoracic hairs. It shows that both the species have a net negative charge (pC) on their bodies after removing the hairs.

Faraday pail charge measurements showed that 97.5% of *C. albipila* flies (n = 40) carried a positive charge. Only one individual was recorded with a negative charge (q_mean_ = 32.8 ± 31.0, SEM = 5 pC). Measured on 40 individuals of *E. tenax*, 92.5% flies were positively charged and 6.5% negatively charged (q_mean_ = 23.0 ± 27.2, SEM = 5 pC). The resulting mean potential change lasted for about 50 ms (Fig. 1a). When the hairs of the specimens were removed, the results were surprising: about 90% (n = 9, total = 10) of the *E. tenax* were negatively charged, and 10% were positively charged, while 80% (n = 8, total = 10) of *C. albipila* were negatively charged and 20% were positively charged (Fig. 1b).

**Figure 2:**
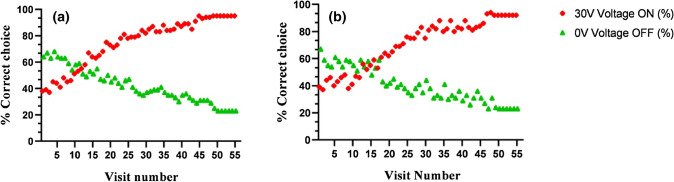
Hoverflies indicates the ability to learn about the presence and absence of an electric field. (**a**) The plot indicates the learning ability of *Cheilosia albipila* to the electric field at 30 V in red and at 0 V (OFF) control in green. In this case, *C. albipila* has access to all three types of flowers (+ ,−and 0) at the same time. (**b**) The graph indicates the learning ability of *Eristalis tenax* to the electric field at 30 V in red and at 0 V (OFF) control in green.

Differential conditionings were used to investigate the ability of hoverflies to distinguish the floral electric field from the background field. The results indicated that flies could identify their flowers of choice in the presence of varying electric fields and that they could remember the location. In a study of visits by 50 flies, charged flowers showed the highest number of visits recorded for both species of hoverfly. The flies’ learning ability was assessed by comparing the mean of the final ten visits (56–65) and a randomly selected choice model. It was noted that in these ten visits to flowers charged to 30 V, *C. albipila* and *E. tenax* achieved 89.5 ± 2.4% and 76.3 ± 2.2% accuracy, respectively (Fig. 2).

**Figure 3:**
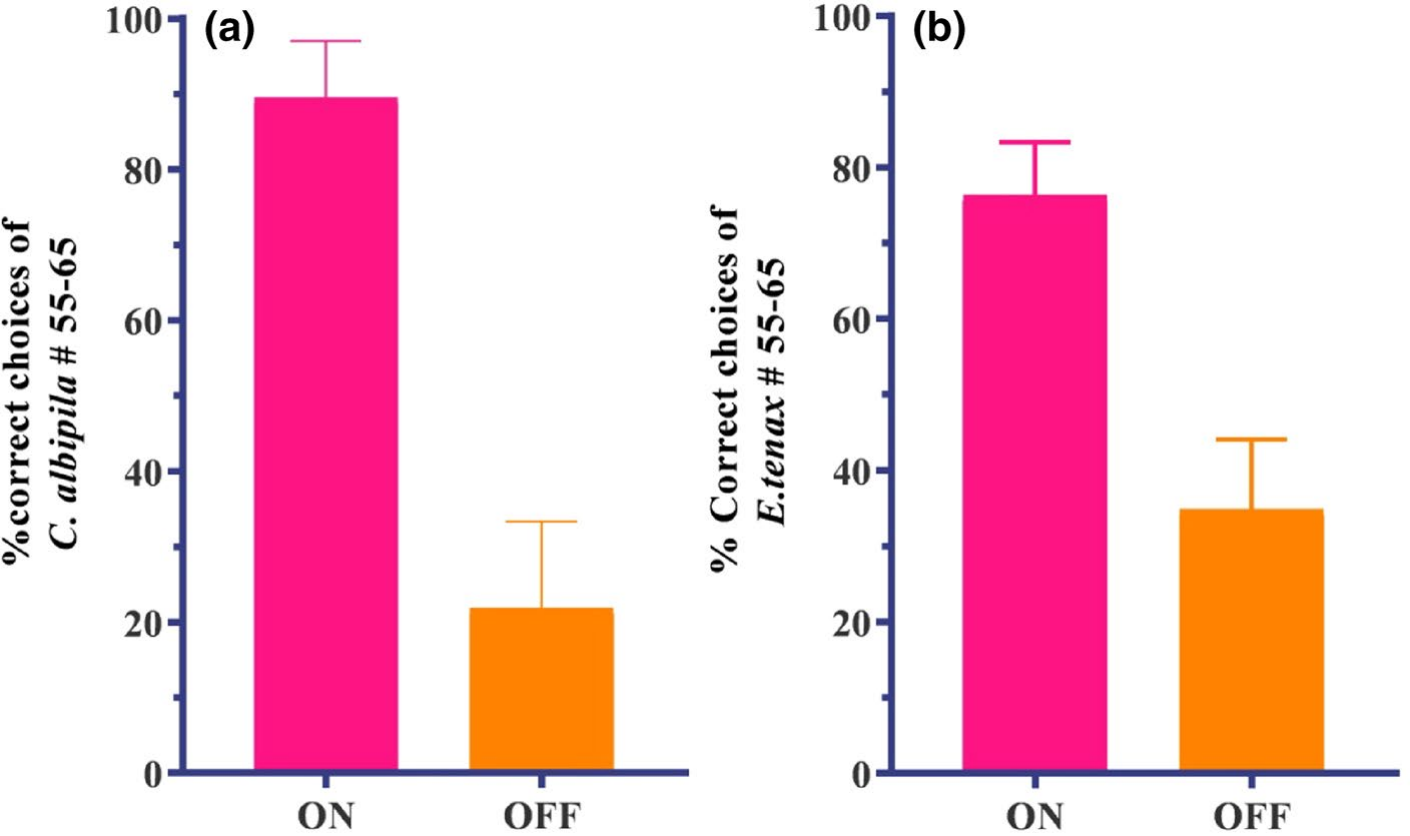
(**a**) Percent of correct choices of *Cheilosia albipila* to last ten visits to rewarding (ON) and non-rewarding (OFF) e-flowers while the error bars show the SEM (Standard error means) (**b**) Percent of correct choices of *Eristalis tenax* to last ten visits to rewarding (ON) and non-rewarding (OFF) e-flowers while the error bars show the SEM.

Both types of flowers were then grounded to 0 V and compared with the choice model. In the absence of electric cues, the same numbers of trained flies were not able to distinguish between rewarding and non-rewarding e-flowers. The highest proportion of correct choices of *C. albipila* in the absence of charge was 64% and the lowest was 23% (mean = 42.2, SD = 13.1, SE = 1.8) (Fig. 2a). In the case of *E. tenax*, after the removal of electric cues (mean = 40.2, SD = 12.7, SE = 1.7), the highest (ON) and lowest (OFF) percent of correct choices were 67% and 23%, respectively (Fig. 2b). Highly significant differences were recorded in the percent of correct choices between charged (30 V, ON) and uncharged (0 V, OFF) flowers for both species (*p* < 0.0001) (Fig. 3a,b). The percent of correct choice in the absence of electric potential dropped from 95 to 65% after switching off the charge and reached 23% at the 50^th^ visit.

Fifty flies of each species were tested and each fly was released three times for three replicates. On average, 34.0 ± 1.7 flies (68%) landed on flowers having the combination of color and electric field, while only 7.7 ± 0.9 flies (16%) were able to identify the flowers by color alone and 8.3 ± 1.7 flies (16%) were able to locate the flowers that only had an electric field.

The motion of sensory hairs in response to an electric field was measured by suspending the pinned flies in front of a laser Doppler vibrometer (LDV). The LDV measured the hair's vibrational velocity, angular displacement, and displacement in response to the electric charge induced on the flies by charged nylon ball bearings. The vibrational velocity (μm/s), displacement (nm), and angular displacement (× 10^−9^) were 53.2 ± 1.05, 2.8 ± 0.27, and 12,757.3 ± 779.9, respectively. The results for *E. tenax* were 52.1 ± 1.1, 2.5 ± 0.3, and 11,571.4 ± 763.9, respectively. The hairs of *C. albipila* responded with significantly greater angular displacement and velocity than those of *E. tenax* (*p* < 0.0007 and *p* < 0.0159, respectively), while the displacement (nm) of hairs of *C. albipila* was not significantly different from *E. tenax* (*p* < 0.9420) (Table S1).

**Figure 4:**
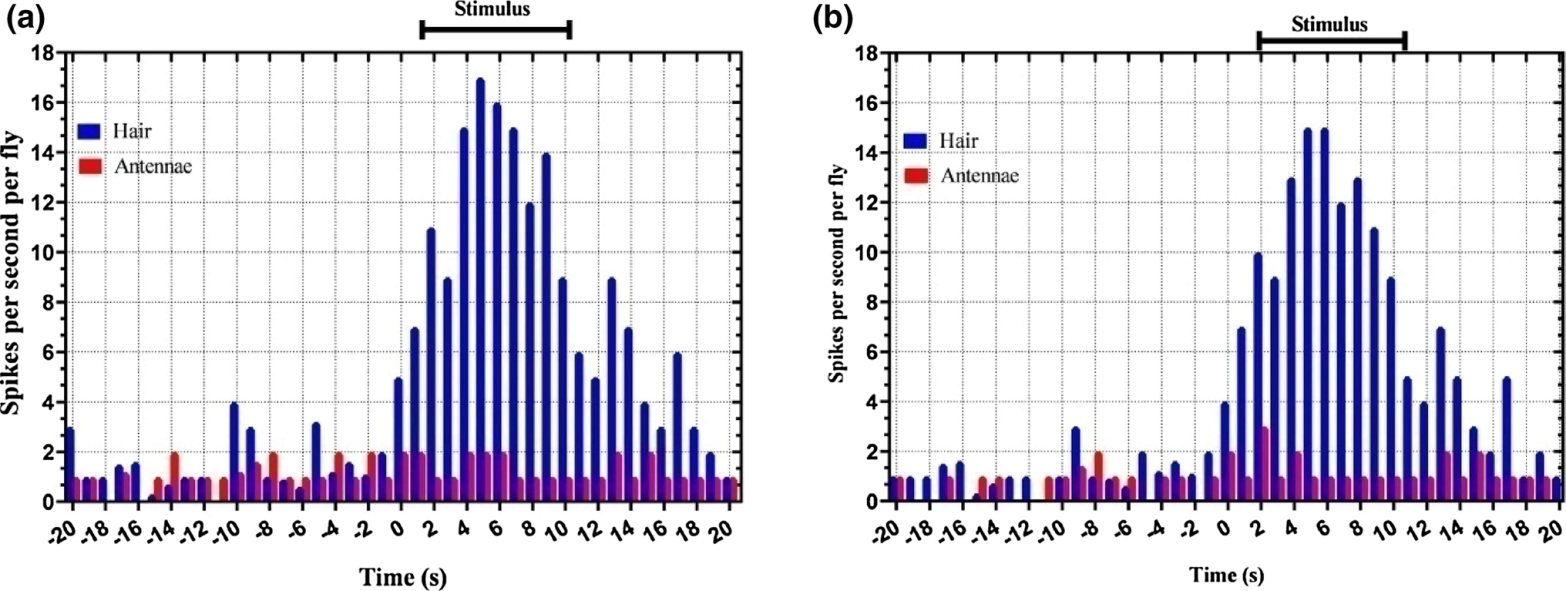
(**a**) The electrophysiological response of antennae (red) and hairs (blue) of *Cheilosia albipila* to a weak electric field applied. The graph indicates the change in rate of firing of nerves of antennae and the hairs to a weak electric field stimulus applied and the blue lines indicate the stimulus. (**b**) The electrophysiological response of antennae (red) and hairs (blue) of *Eristalis tenax* to a weak electric field applied. The graph indicates the change in rate of firing rate of nerves of antennae and the hairs to an electric field stimulus applied and the blue lines indicate the stimulus. The bars represent the numbers of spikes per second per fly.

In the electrophysiological experiments, the thoracic hairs showed increased neural responses to the applied electric field by about 6.8% over non-stimulated hairs (paired *t* test: *p*˂10^–7^). In comparison, there was no increased neural response recorded from the antennae of either species (paired *t* test: *p*˃0.05). Control recordings showed that the antennae of both species responded to air currents and olfactory stimuli (Fig. S1), indicating adequacy of the electrophysiological test (Fig. 4a,b).

The total charge transferred to the artificial flowers by induction from positive charges on the flies was recorded through the stems. The landing of 50 *E. tenax* flies on the flowers induced a mean potential of 15.8 ± 2.2 mV (SD = 15.4, n = 50), while in the case of *C. albipila,* the landing of 37 individuals on the negatively charged flowers induced a potential change of 15.4 ± 2.5 mV (SD = 17.6, n = 50).

## Discussion

Animals from the phylum Arthropoda possess a number of mechanosensory hairs on their bodies for different purposes^32^. Most of these insects use mechanosensory hairs to detect the air currents created by approaching predators^33,34^. Published studies also indicate that cockroaches and honey bees use their antennae to detect electric fields^32,35^. The present study showed that hoverflies used their thoracic hairs to detect the electrostatic charge and electric field around flowers. These results were in line with previous studies conducted on bumblebees, further confirming that insect hairs can be used to detect weak electric fields^36^.

Flowers carry information about previous visits from insects^37^ and their electric potential is directly related to the number of times pollinators have landed on them. Previous visitors significantly affect floral cues by scent or color marks^38,39^ and potential electrostatic charge changes^13^. Electric charges on flowers may last for milliseconds to seconds. Pollinator visits that change floral cues are diverse in nature and reflect their unique behavior. Floral cues also significantly increase the efficiency of pollination^6^ and contribute to a complex behavioral ecology. The amount of opposite charge on the flowers provides information to pollinators about nectar availability, thus influencing their foraging activity and efficiency^13,40^.

Our electrophysiological recordings indicated that the neurons associated with hoverfly thoracic hairs increased neural firing due to the electric field. Laser doppler vibrometry results showed that the thoracic hairs of both fly species, *E. tenax* and *C. albipila*, responded with electrical deflections of 3 × 10^–2^ degs, making them more sensitive than the hairs of bumblebees^36^ that induced deflections of 4 × 10^–2^ but less sensitive than cricket hairs^41^ with deflections on the order of 2 × 10^–2^. A previous study showed that the electrosensory hairs of hoverflies and bumblebees were mechanically and neurophysiologically similar^41^.

We have discovered that the electrostatic field plays an important role in the floral cues of sweet alyssum as it increases the efficiency and speed with which pollinators detect the rewarding resource. Hence, along with vision and olfaction, electric field detection is also an essential source of pollinator floral detection. This discrimination experiment indicates that combining two cues increases a fly’s ability to correctly identify a food source and reject inappropriate targets. As in previous studies, this type of transfer of information between the pollinators and the flowers is rapid, lasting for only a few milliseconds to seconds^5,39^.

## Conclusions

We have discovered that a flower’s electric field acts as a cue for hoverflies. Like other floral cues, the weak electric field increases the speed and accuracy with which the fly finds the nectar reward. We have sought to fill in the gaps in research on hoverflies as this field has received scant attention in spite of their significant contribution to pollination. Like bees, hoverflies make use of electric field sensing along with olfaction and vision. Detection of a weak electric field and its integration into the hoverfly’s sensory ecology needs further exploration. The present study has explored and opened new horizons in understanding the mechanosensory capability of hoverfly thoracic hairs for detecting the weak electric fields of their favorite nectar flowers.

## Materials and methods

### Statements

In the present study, all methods were performed in accordance with relevant guidelines and regulations. There is no need for an ethical statement or license to grow sweet alyssum, *Lobularia maritima* *L.*, in Pakistan.

### Study organisms

The hoverflies, *Eristalis tenax* and *Cheilosia albipila*, are widely distributed throughout Pakistan^42–44^. Specimens were collected from Chakwal (32° 55′ 49″ N 72° 51′ 20″ E), Sargodha (32° 5′ 1″ N 72° 40′ 16″ E) and Murree (33° 54′ 15″ N 73° 23′ 25″ E). After hand-net collection, the specimens were transferred into rearing boxes (box 1 = *E. tenax* and box 2 = *C. albipila*).

The rearing and maintenance of both species was done using previously described methods^45^. The chambers were covered with doubled mosquito nets. Sugar-soaked cotton buds were placed inside the chamber for the adult flies, which preferred to feed on a mixture of glucose, honey, and pollen^46,47^. Adults were kept in line throughout the study period, fed on sucrose solution and a mixture of honey and pollen. The 200 adults of each species (*E. tenax* and *C. albipila*) were released into a large rearing chamber (180 × 180 × 180 cm) containing sweet alyssum and coriander plants.

Sweet alyssum (*Lobularia maritima* *L.*) was selected for the study because hoverflies are readily attracted to their flowers. Sweet alyssum is also prominent in the literature^48^ for attracting non-bee pollinators and is a plant species deemed important for habitat development and pollinator population management. Sweet alyssum attracts more hoverflies than bees for pollination^48^.

### Experimental set-ups

#### Measurements using the Faraday pail

The charge (positive or negative) carried by hoverflies was determined using the JCI 147 Faraday pail and a JCI 140 non-contact voltmeter calibrated as a coulomb meter (Unilab) to measure the voltage on the lower plate, as described in the literature^13,49^. The pail was divided into two plates, the upper finally calibrated capacitor with capacitance (C) While the voltage (V) on the lower plate, which was proportional to the net charge, q, according to the formula q = CV, was then read by the JCI-140^13^. Therefore, the change in voltage on the lower plate when the fly landed on it was equal to the net charge in picocoulombs (pC). Charge measurement was possible because the net inverse charge on the hoverfly was induced on the pail surface (by Faraday electrostatic induction). To measure the charge on the hoverfly species, *E. tenax* and *C. albipila*, specimens were trained to fly free into a Faraday pail that contained a nectar food reward (sucrose solution). The net charge (*q*) on flies was measured from the voltage reading on the capacitor linked to the calibrator^13^. Forty individuals of each species were released (one by one) into the pail and their charge was determined. The results were displayed on the calibrated voltmeter, which indicated the net charge on the object in pC (Fig. 1).

In the second part of the experiment, the thoracic hairs were removed to determine if they acted as electric charge receptor sites. Collected flies of both species were ‘shaved’ by removing the thoracic hairs under an Olympus stereomicroscope (SZX16). The flies were etherized and placed into ‘queen’ holders, which were used to hold queen honey bees during artificial insemination The size of the holders was slightly modified to accommodate the flies. The thoracic part of each fly was outside the holder, and the holders were placed under the microscope. The hairs were removed one by one with a sharp scalpel from the upper and lower portion of the thorax without causing any harm to the flies. Because of the time involved, only ten flies of each species could be ‘shaved’ for each run. After removing the hairs, the Faraday pail experiment was repeated with these flies. A multifunction digital calibrator measured the net opposite charge on the flies.

#### Laser doppler vibrometry

Both hoverfly species (*C. albipila* and *E. tenax*) were freeze killed and attached horizontally to a piece of wood with cyanoacrylate glue on a grounded table^50^. The specimens were attached to stainless steel insect pins (#1) and then placed in the laser doppler vibrometer (LV-1800, ONO SOKKI) to measure movement of the hairs in response to an electrical stimulus. The overhead sensor acquired the data on hair displacement, which was analyzed by displacement analysis software (LV-0930). An electric charge was applied to the suspended flies by contact with a frictionally charged nylon ball bearing and left to settle for 10 min. A 400 Vpp sinusoidal frequency sweep ranging from 10 Hz to 10 kHz for a period of 10 s was delivered to a steel disk at a distance of 1.0 cm from the flies. For each species, ten responses were recorded from two randomly selected hairs. The hair displacement output was acquired by the FFT analyzer, processed by PSV software (Polytec version 9.0), and displayed on the digital output unit, LV-0121A digital laser displacement meter. All the processes and experiments were conducted on a benchtop isolator (auto-leveling LV-0200) to prevent vibration of the stand that was holding the sensor.

### Electrophysiology

Electrophysiology experiments were conducted to investigate the sensory basis of electroreception, by testing the hypothesis that mechanical hair deflections served an electrosensory function. After being anesthetized with CO_2_ (usually less than 10 s for *C. albipila* and less than 20 s for *E. tenax*), the hoverflies were suspended over modeling clay in a vertical position. Electrolytically sharpened tungsten electrodes were used for extracellular recordings from the antennae and hairs. The generated signals were amplified with a WPI DAM-50 high-impedance differential amplifier (World Precision Instruments, Sarasota, Florida) and the outputs were digitized using the EAG 2000 software (Syntech, Hilversum, The Netherlands) and recorded. An experimental electrode was inserted into the antennal scape, and a reference electrode was inserted into the head but not near the eyes to record the antennal response. To record hair responses to the presentation of an electrostatic charge, the recording electrode was inserted into the basal socket while the reference electrode was pinned onto the cuticle membrane of the abdomen.

The electrical stimulus, a 400 Vpp frequency sweep ranging from 10 Hz to 10 kHz for a period of 10 s, was delivered to a steel disk at a 1.0 cm distance from the flies. For all experiments, the flies were held for 10 s with no stimulus, then 10 s of electrical stimulation was applied, and the same process was repeated three times. For the antennae response, an additional air current and A.C. electric current stimulus were applied as described^36^ (Fig. S1).

### Discrimination experiment

Three groups of six artificial black flowers, were placed inside the chamber. One group of artificial flowers had 15% sucrose solution and was connected with the positive terminals, while the other group contained quinine monohydrochloride dihydrate and was negatively charged. The third group of flowers was not connected to either positive or negative terminals. Of the six flowers in the third group, three contained 15% sucrose solution and three had the bitter quinine solution (Fig. S2). The artificial flowers used in this study were 22 mm in diameter and had 1.5-mm-thick bases made of steel and covered with epoxy^13^. One-third of the flowers in this experiment were held at a 30 V DC bias voltage because it was biologically relevant; a typical 30 cm-tall flower can hold up to a 100 Vm^-1^ atmospheric electric field^51^. Charged artificial flowers (e-flowers) contained a sucrose reward while the e-flowers placed at 0 V held bitter quinine hemisulfate solution; e-flowers were identical in all other respects.

To rule out the effect of cues other than electric ones, the following controls were included. The sucrose and quinine solutions cannot be distinguished by scent^52^. All artificial flowers were the same color and separated by 15 cm to prevent induction from a nearby flowers' charge. After each visit, the complete setup was removed and washed with ethanol and water, and the positions of the flowers were randomly changed. Ethanol washing removes any previous ‘footprints’ left by the flies during their visits and any traces of sucrose and quinine. After that, the positions of the flowers were again randomly changed, minimizing the possibility of positional cues. To reduce the chance of electric field generation around the electric wires, electric shielding was used, and the charge was discharged after every visit by grounding, while the digital voltmeter measured the electric potential. For conditioned learning, individuals were released 55 times, and their landings at feeding stations were recorded when the electric field was connected to the grounded flowers. The percent of correct choices of the final ten visits (56–65) was measured against 30 V charged flowers to determine the flies' learning ability.

To test the hypothesis that the electric field of a flower enhanced the effectiveness of the other flower-related cues, an experiment to differentiate among the cues was conducted. Three groups of flowers were placed inside the glass chamber, each containing six flowers with 15% sucrose solution. If the hypothesis is true, then the electric field will positively affect the learning ability of flies. Three groups of artificial flowers were used in the experiment. The first group had a red 0° HSB target hue, the second had an electric charge of 30 V, and the third group of flowers had a red tint along with an electric field on each flower. To test the behavior of the flies in response to the cues, 50 flies were released from the holding chamber, and which flowers they landed on were recorded.

### Measurement of stem electric potential in sweet alyssum during fly landing

**Figure 5:**
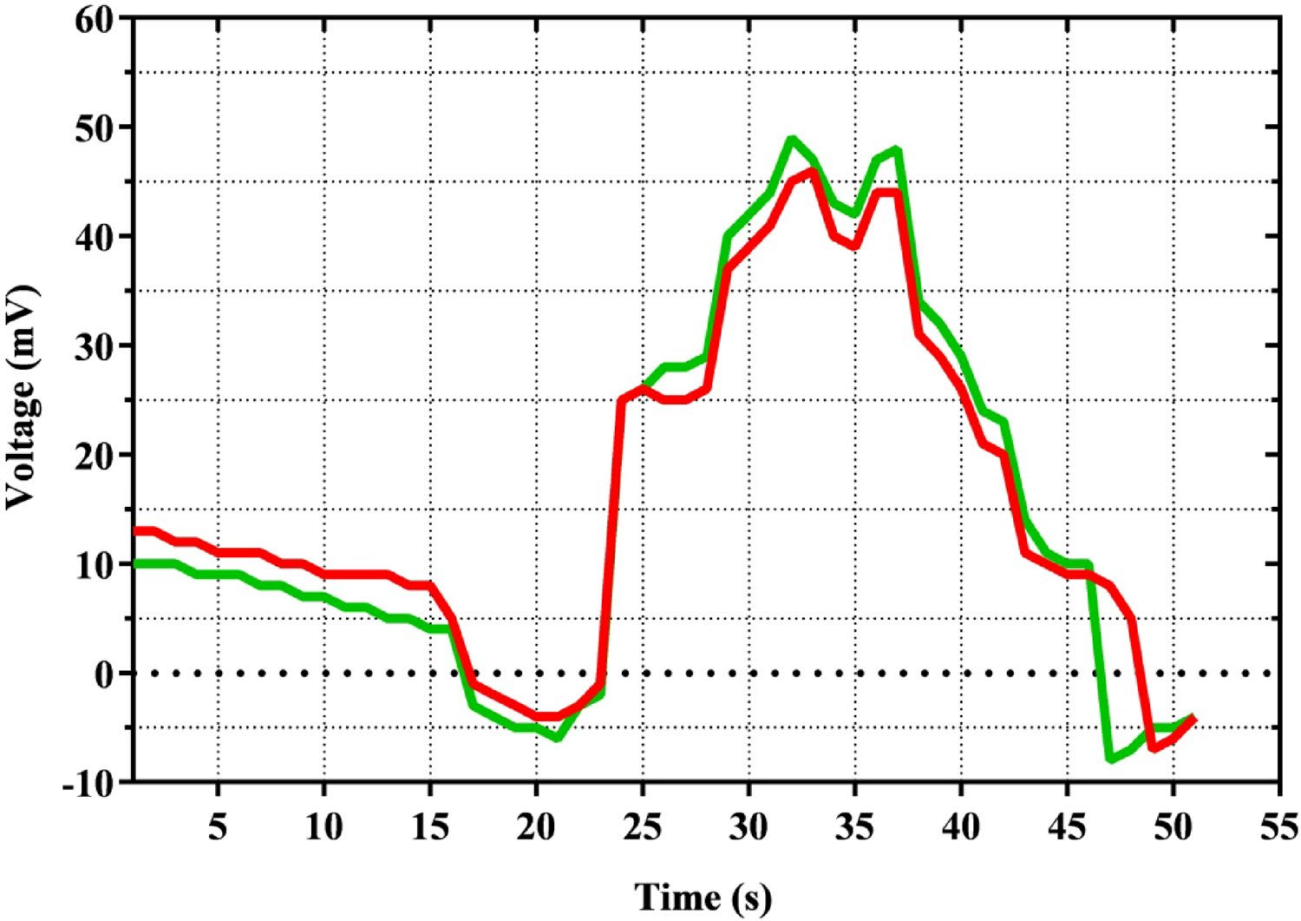
The plot indicates the change in charge in relation to time (s) due to *Eristalis tenax* (red) and *Cheilosia albipila* (green) landing. It is the mean charge variation in relation to the time of flies landed on e-flowers 23 s (*E. tenax*) and 21 s (*C. albipila*) at which the voltage or charge skyrocket.

To measure the electric potential of sweet alyssum, a tungsten electrode was inserted in the stem at the base of the corolla by the method of Stanković and Davies^53^. The DAM-50 Bio-differential amplifier (World Precision Instruments, Sarasota, FL) was used to amplify the electric potential of the stem during the landing of the fly. The electrodes were connected to the amplifier about 3 cm from the flower, and the change in electric potential by the fly landing on the flower was recorded using a data acquisition system (DAQ). The differential method was used to measure the electric potential of the stem, and a reference electrode was inserted into the floral foam for that purpose. The electric potential of the stem was measured by calculating the difference between the measurement electrode and the reference electrode^13^. To allow the flies to land freely on the flower, a 2 × 2 × 2 m Faraday cage was attached to the flies’ holding chamber. The recording of the signals during the landing of each of 50 flies (n = 50) was automatically started when the fly contacted the flower. Before starting an experiment, the response of the measuring electrode was tested by releasing a bumblebee (*Bombus terrestris*) and some house flies (*Musca domestica*) into the chamber. To avoid unwanted movements and torque on the electrode from air currents caused by the fly, a thin layer of floral foam was used. For comparison, twenty recordings of 10 s each were made before the flies were released inside the Faraday cage. In this experiment, first *E. tenax* specimens were released and then *C. albipila*, into the Faraday cage containing flowers of sweet alyssum with the same natural fragrance. The exact process was repeated three times with (n = 50) different individuals of the same species to minimize the chances of error in results (Fig. 5).

### Ethics statement

There is no need for an ethical statement or any other type of license to grow Sweet alyssum (*Lobularia maritima* *L.*) in Pakistan.

## Supplementary Information

Supplementary Information.
