# Supplementary material for: Electric field detection as floral cue in hoverfly pollination
Source: Sci Rep. 2021 Sep 21;11:18781. doi: 10.1038/s41598-021-98371-4 (PMC8455601; doi:10.1038/s41598-021-98371-4)
Supplement: Supplementary file 1 — Supplementary Information. [file 41598_2021_98371_MOESM1_ESM.docx]

**Electric field detection in hoverflies pollinators**

**Shahmshad Ahmed Khan^1^, Khalid Ali Khan^2,3,4^*, Stepan Kubik^5^, Saboor Ahmad^1,6*^, Hamed A. Ghramh^2,3,4^, Afzal Ahmad^7^, Milan Skalicky^8^, Zeenat Naveed^9^, Sadia Malik^10^, Ahlam Khalofah^2,4^**

^1^Laboratory of Apiculture, Department of Entomology, Pir Mehr Ali Shah Arid Agriculture University, Rawalpindi 46000, Pakistan

^2^Research Center for Advanced Materials Science (RCAMS), King Khalid University, P.O. Box 9004 Abha 61413, Saudi Arabia.

^3^Unit of Bee Research and Honey Production, Faculty of Science, King Khalid University, P.O. Box 9004 Abha 61413, Saudi Arabia.

^4^Biology Department, Faculty of Science, King Khalid University, P.O. Box 9004 Abha 61413, Saudi Arabia.

^5^Department of Zoology and Fisheries, Faculty of Agrobiology, Food and Natural Resources, Czech University of Life Sciences Prague, Kamycka 129, 165 00 Praha 6-Suchdol, Czech Republic

^6^Institute of Apicultural Research/Key Laboratory of Pollinating Insect Biology, Ministry of Agriculture, Chinese Academy of Agricultural Sciences, Beijing 100093, China.

^7^Department of Physics, Allama Iqbal Open University, Islamabad 44000, Pakistan

^8^Department of Botany and Plant Physiology, Faculty of Agrobiology, Food and Natural Resources, Czech University of Life Sciences Prague, Kamycka 129, 165 00 Prague, Czechia

^9^Department of Botany, University of Gujarat 50700, Pakistan

^10^Atta ur Rahman School of Applied Biosciences (ASAB), National University of Science and Technology (NUST), Pakistan

*Corresponding author. [saboorahmad86@yahoo.com](mailto:saboorahmad86@yahoo.com)

| 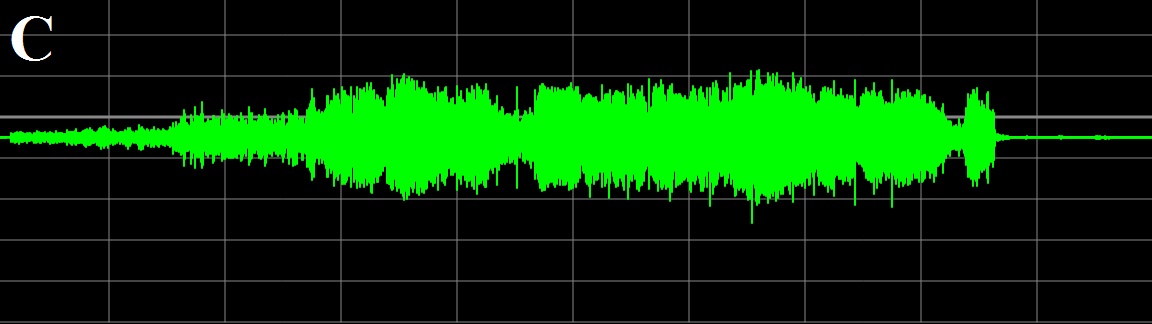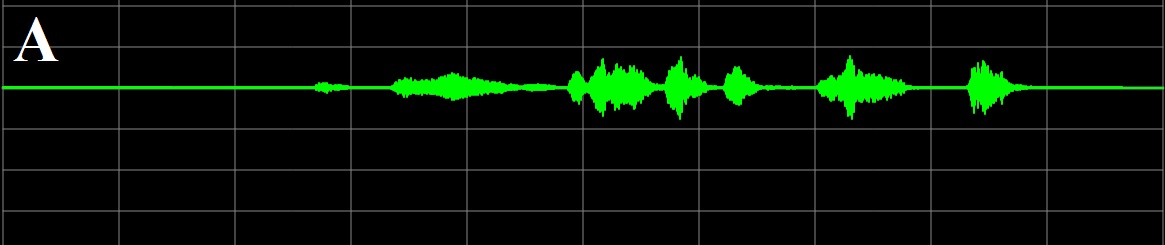 | 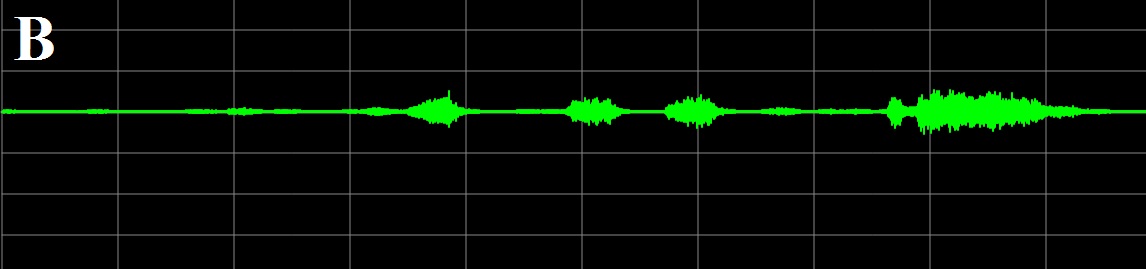 |
| --- | --- |
|  | 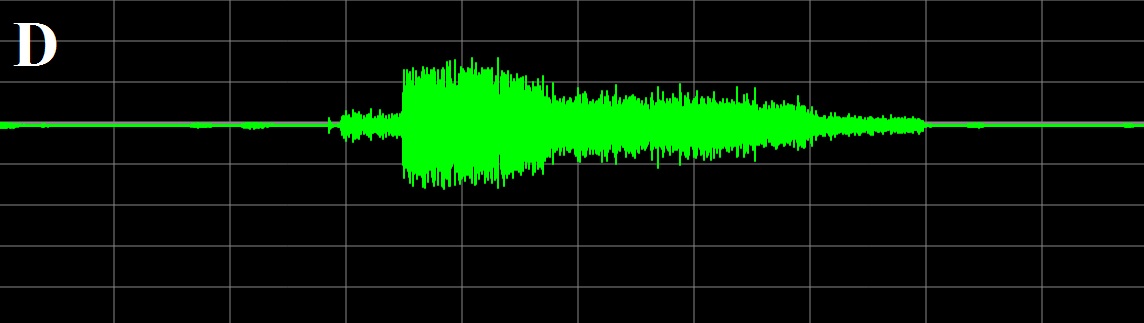 |
| 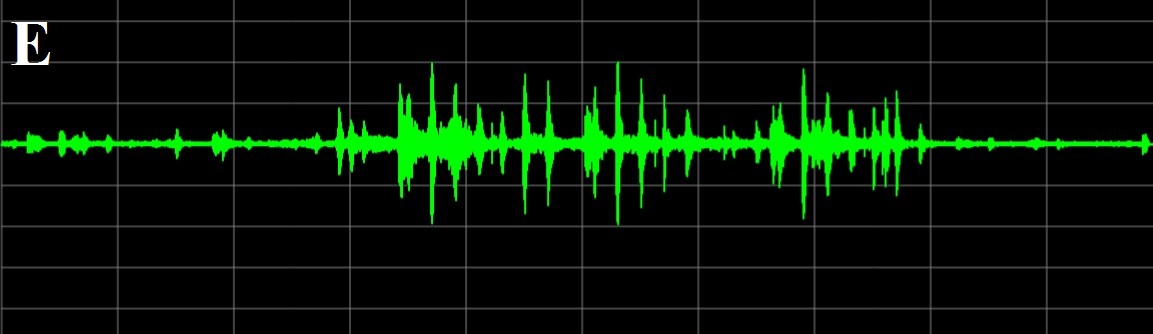 | 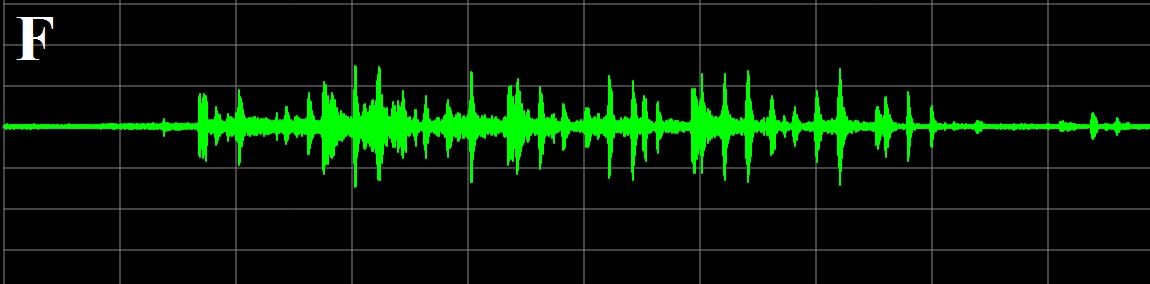 |

**Fig. S1.** Electrophysiological recordings of hair and antennae of both hoverfly species *Cheilosia albipila* and *Eristalis tenax* in response to an electric charge stimulus induced by electrically charged nylon bearing ball. **(A and B)** The antennal response of *C. albipila* and *E. tenax* to an electric stimulus **(C and D)** Hair response of *C. albipila* and *E. tenax* to charge the plot showing the value the numbers of spikes per second, per fly, divided by the mean prestimulus spike rate. **(E and F)** the response of *C. albipila* and *E. tenax* to the antenna a puff of scented air.

**
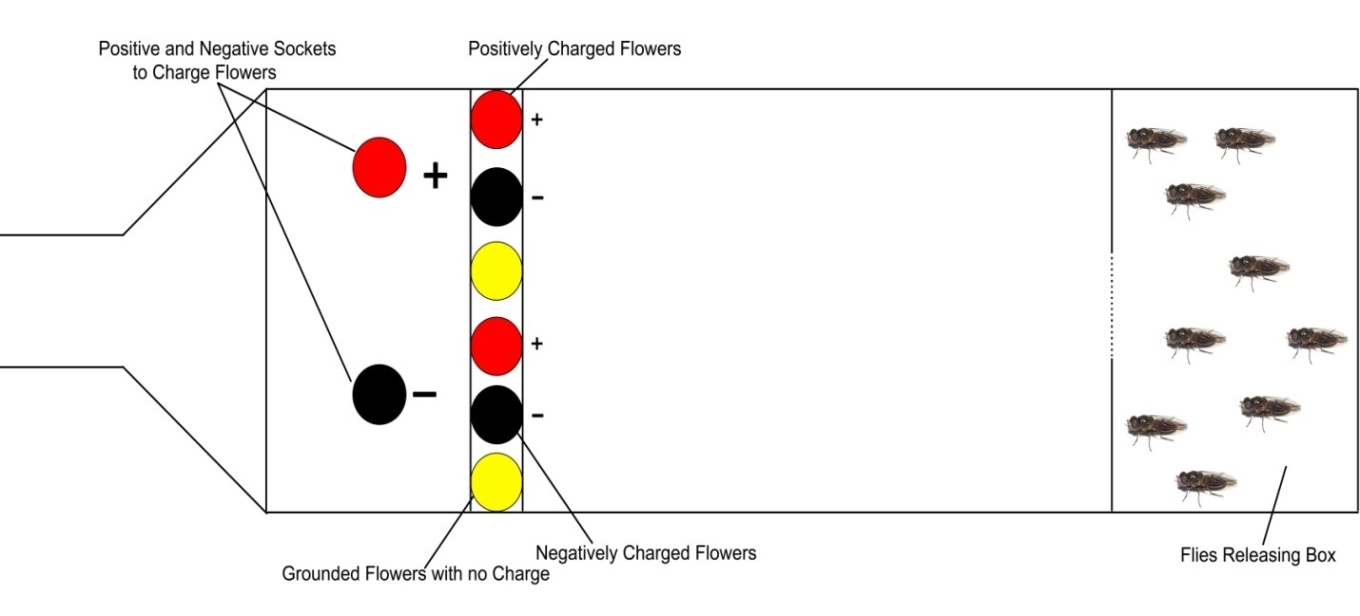
**

**Fig. S2.**Laboratory prepared for the learning of hoverflies. Positive and negative sockets are used for the charging of flowers, an alternate line of positive (Red), negative (Black) and grounded (Yellow) flowers while on the other hand it has releasing/holding the chamber for hoverflies.

**Table S1.** Results of Laser Doppler Vibrometry experiments from *E. tenax* and *C. albipila*

| **Movement** | ***C. albipila*** | ***E. tenax*** | ***Sig.* (*P*)** |
| --- | --- | --- | --- |
| Velocity, μm/s | 53.2±1.05 | 52.058±1.09 | 0.0159 |
| Displacement., nm | 2.8±0.27 | 2.5±0.31 | 0.9420 |
| Angular displacement, degrees × 10^−9^ | 12757.3±779.91 | 11571.4±763.96 | 0.0007 |

It shows the mean value with a standard deviation of hair velocity, displacement, and angular displacement of both species of hoverflies in response to electrical chirps (8.0Hz-12 kHz). The velocity and the angular displacement of the hairs of *C. albipila* are significantly different from the *E. tenax* and non-significant in case of displacement (paired t-test).
